# Identification of lead molecules against potential drug target protein MAPK4 from *L*. *donovani*: An in-silico approach using docking, molecular dynamics and binding free energy calculation

**DOI:** 10.1371/journal.pone.0221331

**Published:** 2019-08-19

**Authors:** Shweta Raj, Santanu Sasidharan, Vikash Kumar Dubey, Prakash Saudagar

**Affiliations:** 1 Department of Biotechnology, National Institute of Technology-Warangal, Warangal, (T.S.), India; 2 School of Biochemical Engineering, Indian Institute of Technology-Banaras Hindu University, Varanasi, Uttar Pradesh, India; Jamia Millia Islamia, INDIA

## Abstract

Leishmaniasis caused by obligate intracellular parasites of genus *Leishmania* is one of the most neglected tropical diseases threatening 350 million people worldwide. Protein kinases have drawn much attention as potential drug targets due to their important role in various cellular processes. In *Leishmania* sp. mitogen-activated protein kinase 4 is essential for the parasite survival because of its involvement in various regulatory, apoptotic and developmental pathways. The current study reveals the identification of natural inhibitors of *L*. *donovani* mitogen-activated protein kinase-4 (LdMPK4). We have performed in silico docking of 110 natural inhibitors of *Leishmania* parasite that have been reported earlier and identified two compounds Genistein (GEN) and Chrysin (CHY). The homology model of LdMPK4 was developed, followed by binding affinity studies, and pharmacokinetic properties of the inhibitors were calculated by maintaining ATP as a standard molecule. The modelled structure was deposited in the protein model database with PMDB ID: PM0080988. Molecular dynamic simulation of the enzyme-inhibitor complex along with the free energy calculations over 50 ns showed that GEN and CHY are more stable in their binding. These two molecules, GEN and CHY, can be considered as lead molecules for targeting LdMPK4 enzyme and could emerge as potential LdMPK4 inhibitors.

## Introduction

Leishmaniases are vector-borne protozoan parasites that belong to the genus *Leishmania* (Kinetoplastida: Trypanosomatidae). Known for their high mortality rates, WHO has estimated 1.3 million new cases and 20,000 deaths every year [1]. The protozoan parasite exists in two forms; the promastigotes that develop in sand-flies (*Phlebotomus sp*.) and the amastigotes that grow and multiply in the macrophages of mammalian hosts. The parasite switches forms as it journeys between the vector and the host [2]. Different species of *Leishmania* causes five types of leishmaniasis and among these, the cutaneous, visceral and mucocutaneous are the most prominent forms studied [3–6]. The cure for the parasitic infection is limited because of the costs involved, efficacy and severe adverse effects, and this has led to reduced treatment options and drug resistance too. The current scenario requires the need for novel and safe drugs and drug target, thereby compelling the need for this study.

There have been previous studies for new targets in *Leishmania sp*. [7, 8]. Among them, protein kinases have been the subject of study for a long time for their role in survival, apoptosis, differentiation and cell responses. More than 500 different protein kinases have been discovered from human genome sequencing [9]. They also play an important role in signal transduction of eukaryotic cells. Kinase family of enzymes phosphorylates other enzymes by adding a phosphate molecule to the amino acid residues of the protein [10]. The event is critical wherein a single phosphoryl molecule resulting from ATP molecule is transferred to the hydroxyl groups of serine, threonine or tyrosine molecules. Most commonly studied kinases are Ser/Thr kinase and followed by Tyr kinase because of their versatile nature in cells.

The Mitogen-Activated Protein Kinases (MAPK) from the Ser/Thr kinase family is yet another long known target for cancer studies and is homologous in diverse organisms including humans. MAPKs play a significant role in the signal transduction cascade by phosphorylating and dephosphorylating kinase enzymes, eventually controlling the expression of protein molecules necessary for cell activities [11, 12]. The genomic sequence of *Leishmania* has identified 15 MAPK genes and they have been found to be homologous in *L*. *mexicana*, *L*. *infantum and L*. *brasiliensis* [13]. Deletion analyses of MAPK genes have shown regulatory problems in parasite development. *Leishmania* MAPKs have been known to carry a long carboxy terminus extension of 52–1186 amino acids and the extension is synonymous even in mammalian MAPKs ERK5 (400 amino acids), ERK7 (195 amino acids) and ERK8 (194 amino acids) [14–17]. The role of extensions in ERK5 and ERK7 has been known to be involved in regulation, cellular localisation, and negative growth regulators respectively but their role in *Leishmania* still remains elusive [18]. Deletion mutants of MAPK3 have also shown shortening of flagella and overexpression of the enzyme with deletion background nullifies this effect [19, 20]. The expression of MAPK4 is essential for both promastigotes and amastigote form of *Leishmania sp*. and the phosphorylation of T190 and Y192 in the phosphorylation lip causes activation of the kinase enzyme [21]. Overexpression of MAPK4 causes stage-specific induction of phosphotransferase activity [22]. MAPK4 from *Leishmania donovani* has very less similarity to mammalian MAP kinases and thereby the enzyme holds a prospective chance of being a unique drug target in *Leishmania*.

**Fig 1:**
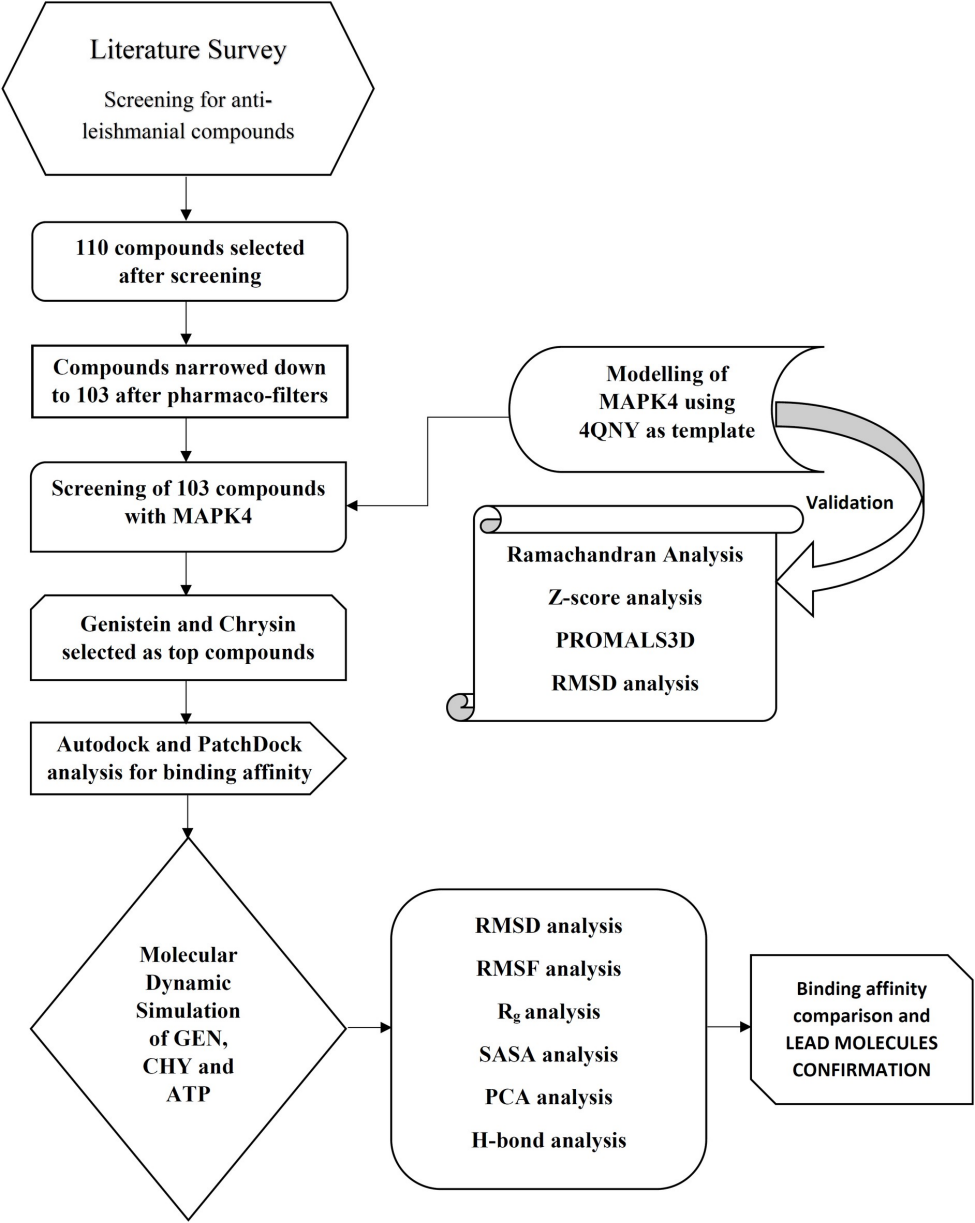
Workflow. Schematic representation of workflow for inhibitor screening, docking and simulation.

The workflow model for the study is depicted in Fig 1. The lack of crystal structure of *Leishmania* MAPK4 pushed us to perform homology modelling of *Leishmania donovani* MAPK4 (LdMPK4) by utilising the MAPK3 crystal structure from *Leishmania donovani*. The similarity of being from the same species allowed us to derive the modelled structure and structural domains of LdMPK4. The modelled structure was validated using various web servers and was found to be fit for further study. The stability of the enzyme was also tested using dynamic simulation over 100 nanoseconds (ns). Screening and docking for various natural inhibitors were also performed while maintaining ATP molecule as a positive control throughout. Two compounds genistein (GEN) and chrysin (CHY) were found to have good binding affinities and were investigated additionally to satisfy drug-likeliness. Subsequently, the binding affinities and stability of the enzyme-inhibitor complexes were put to test using molecular dynamic simulations for over 50 ns and binding free energies were determined. From this study, we report the identification of natural lead compounds GEN and CHY as potential inhibitors against LdMPK4. These novel compounds GEN and CHY reported in the study can be further evaluated for their anti-leishmanial activity and can be developed into drugs against leishmaniasis.

## Materials and methods

### Template selection

The lack of solved structures of LdMPK4 was assessed and homology modelling was opted to determine the structure of LdMPK4. The sequence of LdMPK4 was retrieved from the NCBI protein database and PSI-BLAST[23] was performed against Protein Data Bank. Several structures were retrieved and analysed for resolution, R-value, Ramachandran outliers and ligand complexed. Sequence similarity of the retrieved structures were performed using MUSCLE 3.8 [24]. Among these, *Leishmania donovani* MAPK (PDB ID: 4QNY) was considered, for the reason that the resolution was 2.71 Å and that it shares 54% similarity and 39% identity with LdMPK4. The structure houses a structurally similar ligand Phosphoaminophosphonic acid-adenylate ester, thus confirming the intactness of the binding pocket. PDB ID: 4QNY was thus selected for homology modelling of LdMPK4.

### Homology modelling and structure validation

The putative gene sequence of LdMPK4 consisting of 1.16 kb was retrieved from the NCBI nucleotide database (Gene id: 13386132) and the protein sequence was obtained from UniprotKB Database (Accession id: Q9U6V4). Structural and sequential data for PDB ID: 4QNY was also retrieved from Protein Data Bank. For comparative modelling of LdMPK4, Modeller v9.19 [25] was chosen and multiple sequence alignment was performed to build the tertiary structure model. Over 50 3D homology models were produced and the validation for the best model was done using GA341[26] and DOPE [27] scores. To structurally validate the models, the structures were analysed by SAVES validation package [28, 29] and RAMPAGE server (http://mordred.bioc.cam.ac.uk/~rapper/rampage.php). ProSA program was also used to analyse the quality of the best model along with the template [30]. Model-template superimposition was performed using Chimera molecular visualization program [31]. The best model was energy minimised using GROMACS package [32]. The model was minimised by Amber 99 force field and utilising steepest gradient algorithm. The lowest potential energy attained modelled structure was then deposited in Protein Model Database [33] and PMDB ID: PM0080988 was assigned to the model.

### Binding site prediction

The domain and functional sites of PM0080988 was identified using PROSITE server [34]. The binding site residues of the model within the functional domain were predicted using COACH server [35] and the redundant results were analysed and checked. Superimposition of crystal structures of diverse MAPKs were performed using Chimera molecular visualisation tool and the binding site was compared. The sequence alignment of diverse MAPKs from Human (PDB ID: 5MTX), *Plasmodium berghei* (PDB ID: 3N9X), *Leishmania donovani* (PDB ID: 4QNY) and *Toxoplasma gondii* (PDB ID: 3RP9) along with PM0080988 (s001) was performed using BLOSUM62 [36] and secondary structure predictions by PSIPRED [37] were obtained from PROMALS3D [38].

### Ligand property prediction

The pharmaceutical properties along with ADME properties of the identified ligands selected for docking were assessed using FAFDrugs4 [39]. The pharmacokinetics, drug-likeliness and medicinal chemistry of the chosen compounds were also predicted using SwissADME [40]. Satisfaction of filters like Lipinski’s rule of five [41], Ghose [42], Veber (GSK) [43], Egan (Pharmacia) [44] and Muegge (Bayer) [45] were kept mandatory for further studies. Out of 110 ligands chosen, 7 ligands did not meet the pharmaco-filter requirements and therefore were ruled out.

### Preparation and screening of ligands

Preparation and screening of ligands were performed using PyRX [46]. The 103 ligands were chosen based on previous literature and the structures were retrieved from PubChem database https://pubchem.ncbi.nlm.nih.gov/. PM0080988 was used in this study as a macromolecule for the screening of the ligands. The ligands were energy minimised by utilising conjugate gradient algorithm and Universal Force Field (UFF) [47] and the total number of steps was set to 500. Kollman charge and gasteiger charge were assigned to the PM0080988 and ligands respectively. After minimisation of ligand molecules to the lowest potential energy, the Autodock wizard of PyRx was run using Lamarckian genetic algorithm (LGA) for small molecule conformational search over macromolecule surface. The results of 50 LGA runs for each ligand were ranked based upon their binding affinity and the top 5 result ligand structure were retrieved for further docking analysis and specific interaction with the active site residues of the protein.

### Molecular docking

Autodock v4.2 [48] was considered for docking analysis and interaction studies. The top 5 ligands were docked against PM0080988 macromolecule using Autodock. The polar hydrogens were added and grid maps were assigned using AutoGrid utility with 54 x 64 x 64 points to cover the residues present in the binding cavity. The grid spacing was set to 0.375 Å. All the grid maps were generated for protein and ligands along with electrostatic and desolvation maps. The docking was performed with an initial population size of 150 and was conducted for 200 LGA runs. The results were ranked based on their free energy of binding, inhibition constant and hydrogen bond formed with the residues. The top poses were visually analysed for hydrogen bonding and van der Waals interaction with the enzyme and chemical geometry of the poses were found to be good. Docking of ATP molecule with PM0080988 was also performed to analyse the residues involved in interactions. The molecules were ranked based on their binding energy differences and hydrogen bonds and the top two molecules with the lowest binding energies were carried forward for molecular dynamic simulation. For comparison of docking results with Autodock, all ligands were docked with PM0080988 using Patchdock server [49].

### Molecular dynamic simulation

The MD simulation of the top 2 docked complexes and ATP complex were carried out using Gromacs package. The protein PM0080988 was set for MD simulation under Amber99 force field. The ligand topologies and parameter files were generated using ACPYPE [50] and they were checked for errors manually. The protein-ligand complexes were solvated by TIP4 (Transferable Intermolecular Potential with 4 points) water molecules within a dodecahedron box maintaining a distance of 10 Å between the box edges and the protein surface. The system was neutralised using 3 NA^+^ ions and was energy minimised using steepest descent integrator. The three models were then subjected to position restrained canonically-defined NVT (constant number of atoms N, Volume V, and Temperature T) and NPT (constant number of atoms N, Pressure P and Temperature T) ensemble for 5 ns. Parrinello-Rahman and Modified Berendsen thermostat were used in NPT and NVT equilibration respectively. The models were equilibrated for NVT and NPT at 300 k and 1 bar respectively. The stability of PM0080988 was initially analysed for 100 ns and after verifying the same the simulation of protein-ligand complexes was carried out for 50 ns for comparative trajectory analysis. The secondary structure of PM0080988 was also computed using DSSP v2.0 [51] for the 100 ns using the trajectory file. The free energy of binding and the energy contributed by the residues for non-binding interactions were calculated using g_mmpbsa (Molecular Mechanics Poisson-Boltzmann Surface Area) Gromacs tool [52]. The trajectory analyses were initiated in Gromacs and the graphical analyses was carried out in Xmgrace.

## Results and discussion

### Modelling and structure validation

**Fig 2:**
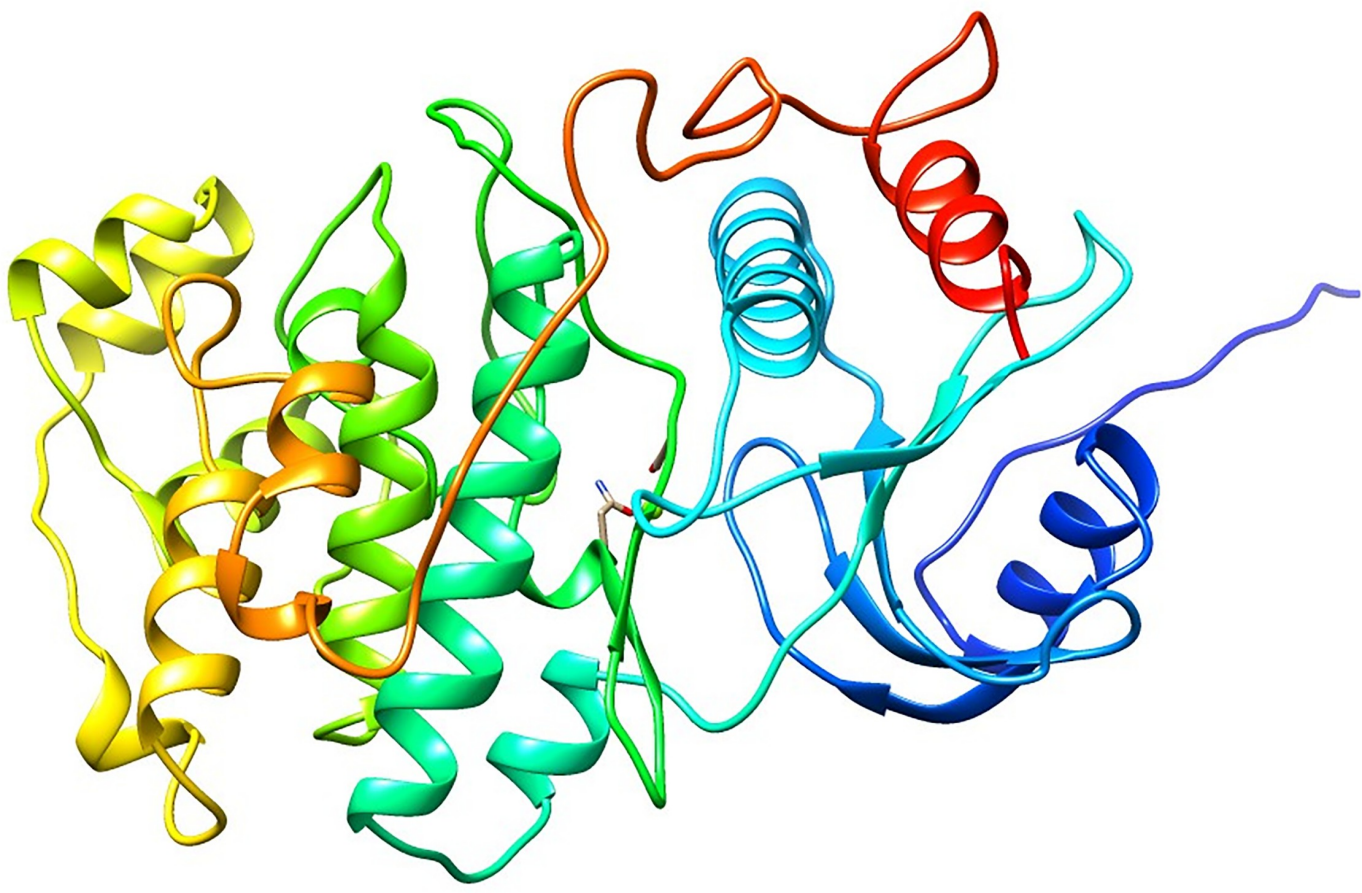
Structure of PM0080988. The α-helix rich structure of LdMPK4 is depicted in the figure. The N-terminal domain is blue and C-terminal domain is represented in red.

The modelled structure of the LdMPK4 was generated using crystal structure 4QNY as the template. Out of 25 models generated, the top 5 models were selected based on the GA341 value of about 1 and DOPE score. The top 5 models were checked for stereochemistry using Ramachandran plot from RAMPAGE. The best model i.e. Model-2 with DOPE score -40796.56 had 92.5% of amino acid residues in the favoured region, 5.8% of residues in the allowed region and 1.7% residues in the outlier region (S1A Fig). The Verify3D results showed that 79.34% of the residues have an averaged 3D-1D score greater than or equal to 0.2. The errat quality plot of Model-2 was found to be within the 95% confidence limit for the binding cavity residues. The quality of Model-2 was assessed from the z-score from ProSa structure validation program. The graphical plot consisting of scores of all the experimentally determined X-ray and NMR protein structures from different sources was utilised to compare the quality of Model-2. The z-score was obtained as -6.46 which lies in the score range of native proteins of similar size (S1B Fig). Multiple sequence alignment and secondary structure prediction (PSIPRED) by PROMALS3D is depicted in S2 Fig. The active site residue Lys59 and active site residues were found to be conserved throughout different organisms. The RMSD value of Model-2 with the 4QNY template was calculated and was found to be 8.27 Å. Model-2 was then deposited in the protein model database with PMDB ID: PM0080988. The structure of PM0080988 predominantly contains α-helix and coils. The conserved binding site amino acid Lys59 is located in a cavity that houses a Mg ion also. The N-terminal domain of LdMPK4 is a coil that stretches out while the C-terminal is composed of α-helix. The structure of PM0080988 is represented in Fig 2.

### Binding site prediction and ligand validation

Binding site production using COACH identified ATP binding sites as Gly37, Phe38, Gly39, Cys41, Gly42, Lys59, Arg73, Glu77, Met81, Tyr114, Asp115, Thr116, Asp117, Arg120, Ser160, Asn161, Leu163, Cys173, Asp174, and Phe175. These residues were found to be conserved in all the kinases that were assessed. PROSITE server predicted Lys59 as the active site residue forming bonds with the phosphate group of ATP. The comparison displayed active site residue conservation in the ATP binding cavity. 103 ligands selected were taken for docking using the grid selection of the active cavity. The screening allowed us to identify GEN and CHY as the best ligands for further docking studies and molecular simulation. The binding energy affinity using PyRx was found to be -6.57 kcal/mol and -6.89 kcal/mol for GEN and CHY respectively. The molecules were selected based on their affinity to affect tyrosine kinases and cancer cell lines.

GEN molecule is an isoflavone that has been found to be an angiogenesis inhibitor and a phytoestrogen. Isoflavones are a class of phytoestrogens known to exhibit estrogenic and antioxidant properties. Isolated from *Genista tinctoria*, the molecule has 15 carbon atoms and a molecular weight of 270.24g/mol. The molecule has three hydroxyl groups which are capable of hydrogen bond formation. The molecule has also been found to have links with cancer and various other anti-properties [53]. GEN has been reported to ameliorate colitis by polarization of M1 macrophages to M2 phenotype and helps reduce the pro-inflammatory cytokine [54]. GEN also suppresses LPS-induced inflammatory response by inhibiting NF-ĸB in macrophages [55]. There is uptake of naturally occurring isoflavone genistein naturally by macrophages and therefore, the parasites residing in the macrophages will be susceptible to the GEN compound. CHY is a flavone with anti-proliferative activity in cancer cells. Flavones are flavonoid types that have been found in honey, mushrooms, and carrots. CHY molecule is found in abundance in honey propolis, honeycomb, and consists of 15 carbon molecule with 254.24 g/mol molecular weight. CHY molecule has two hydroxyl groups attached to its cyclic structure that are capable of forming hydrogen bonds. The presence of hydroxyl groups in GEN and CHY makes it a potential drug molecule capable of hydrogen bond formation in the active site cavity.

The chance of failure of a ligand molecule during clinical trials can be evaluated in prior *in-silico*. GEN and CHY were also validated using FAFDrugs4 and SwissADME server for their pharmacological and pharmacokinetic properties (Table 1). The compliance with Lipinski’s rule of five and satisfaction of other filters suggested the favourable pharmacological side of the molecules. The chemical structure of GEN and CHY molecules are represented in S3 Fig. The values obtained were well within the range which thereby suggested the concrete eligibility and bioavailability of GEN and CHY.

**Table 1:** Physiochemical prediction. Physiochemical prediction of GEN and CHY ligands as predicted by Swiss ADME and FAFDrugs4 server.

Property/Descriptor & Recommended Values	GEN	CHY
Molecular Weight (130–725)	270.24	254.24
HB Donor (0.6–6.0)	3	2
HB Acceptor (2.0–20.0)	5	4
Rotatable Bonds (0–15)	1	1
TPSA (7–200)	90.90 Å	70.67
Solubility	Moderate	Moderate
GI Absorption	High	High
Log P_o/w_ (-2.0–6.5)	2.04	2.55
Violation of Lipinski, Ghose, Veber, Egan, Muegge rules	No	No

### Binding mode analysis and intermolecular interaction of GEN and CHY

Rigid docking performed using Autodock provided an insight into the binding affinity and hydrogen bond formation of the ligands on the kinase enzyme. To substantiate the binding activity of the inhibitors, the docking was also confirmed using PatchDock server. The inhibitors were both allowed to dock at the ATP binding site. GEN molecule displayed binding energy of -6.57 kcal/mol and the intermolecular energy contributed -7.76 kcal/mol to the binding energy. CHY’s binding energy was calculated to be -6.32 kcal/mol and the intermolecular energy was found to be -7.21 kcal/mol. Both the inhibitors had better binding energy than ATP molecule (-5.48 kcal/mol). The inhibition constants of the ATP, GEN and CHY were examined to be 96.38 μM, 15.25 μM, and 23.45 μM respectively. Inhibition constant (K_i_) is inversely proportional to the binding affinity of the ligand to the enzyme. The hydrogen bond formation was analysed for each molecule to see the contribution of each molecule to the ATP binding sites.

ATP formed hydrogen bonds with Phe38 (2.12 Å), Lys59 (2.12 Å), Glu77 (2.02 Å and 1.72 Å) and Ser160 (1.9 Å, 1.93 Å, and 2.04 Å). The N18 atom of adenosine in ATP bonded to Phe38 while γ phosphate group formed two hydrogen bonds with Glu77. The 5^th^ and 6^th^ OH group in the ribose group of ATP bonded to Ser160 along with the N19 of adenosine which also was bonded to Ser160. The γ phosphate group of the ATP was found to be aligned at a distance of 2.12 Å from the Lys59 active residue. These interactions were found to be in agreement with the active site residues predicted using COACH server.

On the other hand, GEN hydrogen bond formation was found to be with Lys59 (1.97 Å), Arg60 (2.24 Å) and Ser160 (1.8 Å). The hydroxyl group at the 4^th^ position of the 1^st^ cyclic ring bonded to the Arg60 residue. The 5^th^ hydroxyl group on the 3^rd^ ring of GEN molecule formed hydrogen bond with Ser180 amino acid residue. The OH group at the 2^nd^ position of the 1^st^ cyclic ring was found to form a hydrogen bond with the active Lys59 residue with a distance of 1.97 Å. All the interactions with the predicted active residues helped the ligand GEN stabilize within the binding cavity.

**Fig 3:**
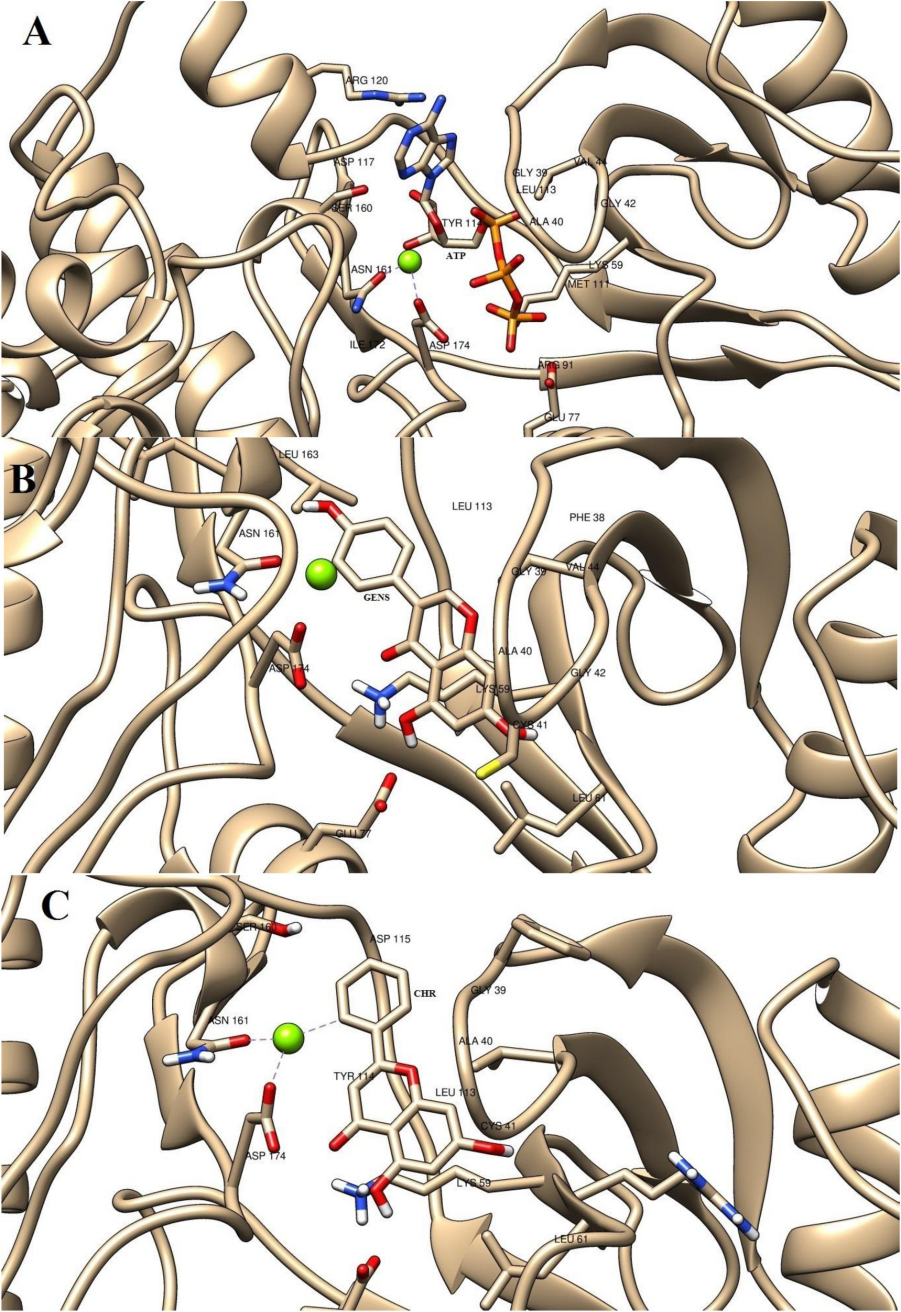
Docked poses of the molecules. Schematic representation of docked protein-ligand interaction where LdMPK4 interaction with (A) ATP, (B) GEN and (C) CHY is shown. All the docking shown was carried out in ATP binding site.

CHY natural molecule with a binding energy of -6.32 kcal/mol formed hydrogen bonds with Arg73 (1.82 Å), Asn161 (2.00 Å) and Asp174 (1.83 Å). The hydroxyl group present in the 1^st^ ring bonded to the Arg73 while the hydroxyl group in the meta position of the same ring formed hydrogen bond with Asp174. The hydroxyl group in the second ring of the CHY molecule bonded to the Asn161 residue of the kinase enzyme. The active site residue Lys59 was not involved in the hydrogen bonding and upon analysis was at a distance greater than 3.5 Å in all the docked conformers. Even though the active site was not involved in the hydrogen bonding, the active site residues were involved in the binding cavity thus blocking the binding cavity. The AutoDock binding free energy calculations for GEN, ATP, and CHY is tabulated in Table 2. Comparative analysis of ligand docked LdMPK4 active site is shown in Fig 3.

**Table 2:** Binding energy calculation. Binding energies and other energy components of MAPK complexes with ATP, GEN and CHY derived from Autodock.

Ligand	Binding Energy (kcal/mol)	Intermolecular energy (kcal/mol)	Internal energy (Kcal/mol)	Unbound energy (kcal/mol)	Torsional energy (kcal/mol)	Inhibition Constant (μM)	No. of Hydrogen Bonds
ATP	-5.48	-9.95	-1.06	-1.06	4.47	96.38	7
GEN	-6.57	-7.76	-0.23	-0.23	1.19	15.25	3
CHY	-6.32	-7.21	-0.96	-0.96	0.89	23.45	3

PatchDock server analysis found that GEN and CHY had Atomic Contact Energy (ACE) as -176.98 kcal/mol and -179.17 kcal/mol (Table 3). These energies were found to be significantly different from ATP ACE -101.34 kcal/mol. The significant difference in the binding energy and molecular interactions of GEN and CHY from ATP in AutoDock and PatchDock suggest that the molecules find the ATP binding site a structurally and energetically favourable place to bind to. The formation of hydrogen bonds with the active site residues by GEN and CHY along with better K_i_ values when compared to ATP suggests that the GEN and CHY are potential inhibitor compounds. The representation of superimposed ATP, GEN, and CHY sharing the same binding cavity is illustrated in S4 Fig.

**Table 3:** PatchDock ACE results of MAPK complexes with ATP, GEN and CHY.

Ligand	Area	ACE(kcal/mol)
ATP	603.50	-101.34
GEN	463.10	-176.98
CHY	477.00	-179.17

### Molecular dynamic simulation of GEN and CHY docked complexes

#### RMSD analysis and secondary structure prediction

**Fig 4:**
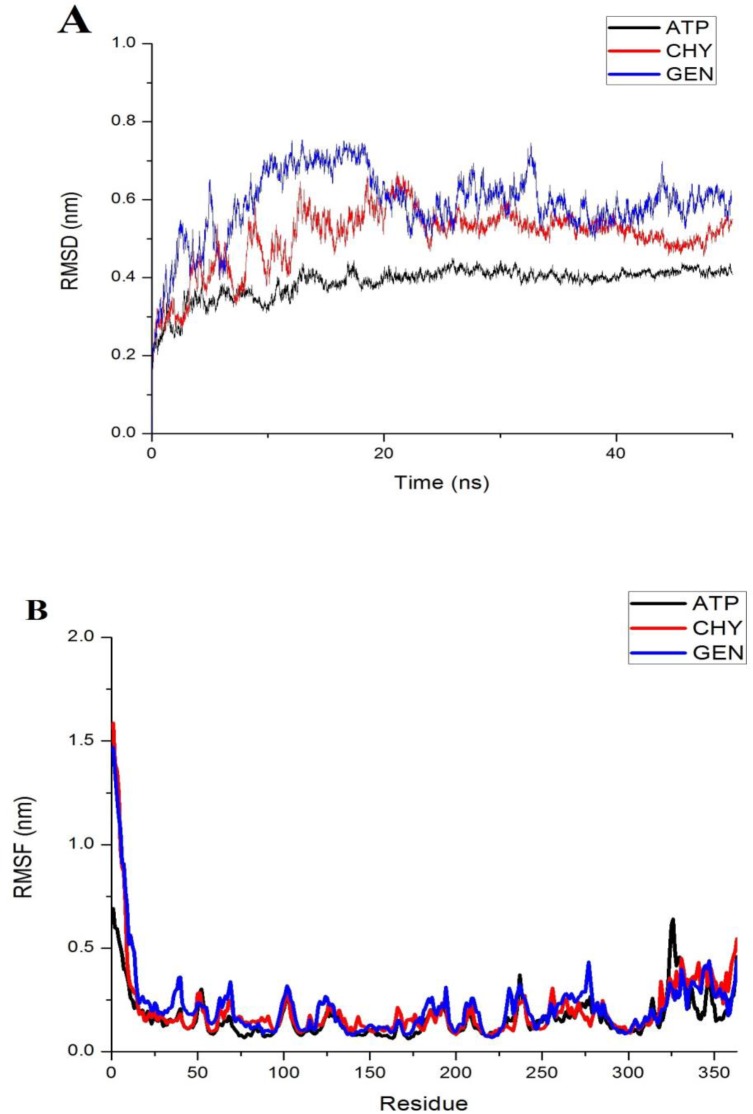
RMSD and RMSF analysis. Analysis of ATP, GEN and CHY simulated protein complex. (A) Time dependent RMSD analysis of ligand ATP(black), CHY(red) and GEN(blue) protein backbone. (B) RMSF analysis of 363 amino acid residues is represented for ATP(black), CHY(red) and GEN(blue) protein complexes.

The molecular simulation and dynamics were carried out to understand the stability of the docked complexes. ATP docked complex simulation was also carried out for comparative analysis. Root Mean Square Deviation (RMSD) graphs were generated with respect to time during the 50 ns production relative to the backbone of the initial structure of the modelled LdMPK4. Average RMSD of apo-LdMPK4 was analysed and found to be 0.4525 ± 0.0733 nm. The average RMSD for backbone atoms of the protein-ligand complex was calculated to be 0.9878±0.0415 nm for ATP complex where the RMSD gradually increased till 10 ns and converged from 20 ns. There were no fluctuations observed thereon. In the case of GEN RMSD analysis, the average RMSD was found to be 0.5040±0.07855 nm. The RMSD value gained till 10 ns and the convergence was observed from 20 ns but minor fluctuations remained throughout. For CHY complex, average RMSD for backbone protein was calculated as 0.5901±0.0873 nm with the values increasing till 12–14 ns and converging from 22–24 ns. No large fluctuations were observed over the rest of the period to 50 ns. The superimposed graphical representation of the time-dependent RMSD of ATP, GEN and CHY are represented in Fig 4A. gmx do_dssp (Dictionary of Secondary Structure of Proteins) assignment of the secondary structure of the apo-LdMPK4 enzyme suggested that the active site residues lie in the coil region and most of the binding cavity residues formed β-helix structure. The predicted secondary structure remained stable for the 100 ns simulation period with insignificant variations in between. The time-dependent secondary structure prediction for 60 ns is represented in S5 Fig. Most of the other residues followed stable structural conformations with very few residues choosing to switch from bends to turns conformation. The prediction recommends the stability of active site residues and the total protein structure over the simulation time period.

#### RMSF analysis

Residue-based Root Mean Square Fluctuations (RMSF) analysis of 363 total residues in LdMPK4 complexes with ATP, GEN and CHY were determined to understand the flexibility of each residue and superimposed graphical representation is shown in Fig 4B. The magnitude of fluctuation of each of the residues is calculated by RMSF values. The RMSF of all the three complexes had very large fluctuation ranging from 0.25–1.6 nm for the first 10 residues. The fluctuation is due to the non-availability of structural information for the modelled protein. The template that was chosen did not have any similarity for this region and so the region remained without any structure. The binding site and the active site residues predicted by COACH server were all found to have lesser fluctuation for all the ligand-enzyme complexes indicating intactness and rigidity of the binding cavity. The superimposition of RMSF values of ATP, GEN and CHY complexed LdMPK4 also indicated that synonymous fluctuation patterns for most of the residues throughout.

#### Radius of gyration

Compactness and structural changes of the ATP, GEN, and CHY complex were reviewed using R_g_ value calculate by gmx gyrate. The radius of gyration measures the mass of the atoms with relation to the center of mass of the complex protein. ATP complex displayed an average R_g_ value of 2.36 ± 0.19 nm and did not show any deviation after 5 ns. The average R_g_ value of 2.29 ± 0.05 nm was observed over the simulation period of 50 ns in GEN-LdMPK4 complex whereas it was 2.39 ± 0.10 nm in CHY complex. Both the complex remained intact after 5–7 ns time period. The structural compactness and compactness of the inhibitor-protein complex were similar to that of ATP complex. It was also observed that the R_g_ values correlated well to the RMSD values of backbone Cα atoms. The difference between ATP complex and inhibitor-complexed LdMPK4 being insignificant suggests that the ligand-protein complexes remained relatively stable after a period of 7 ns and also indicated the stable folding of the modelled protein.

#### SASA analysis

Solvent accessible surface area (SASA) was calculated and analysed for the three ligand-protein complexes to understand the interaction between the complex and the solvents. Conformational changes in the protein structure upon binding of different ligands were predicted using gmx sasa. The binding affinity of the inhibitors to the protein can be studied by desolvation of the protein cavity, ligand and residue rearrangement. The average SASA value of ATP complex was calculated as 200 ± 6.5 nm over the period of 50 ns. GEN complex produced an average SASA value of 202.31 ± 5.29 nm whereas CHY averaged SASA value was calculated as 202.84 ± 4.15 nm. Even though ATP and CHY bound protein had fluctuations over the initial period, GEN complex showed very little fluctuation. Throughout the production time, the binding of ATP and inhibitors to the LdMPK4 induced very little conformational changes in the protein. The results of the SASA analysis indicated that the active site residues are well exposed to the solvent and readily accessible.

#### Principal component analysis

**Fig 5:**
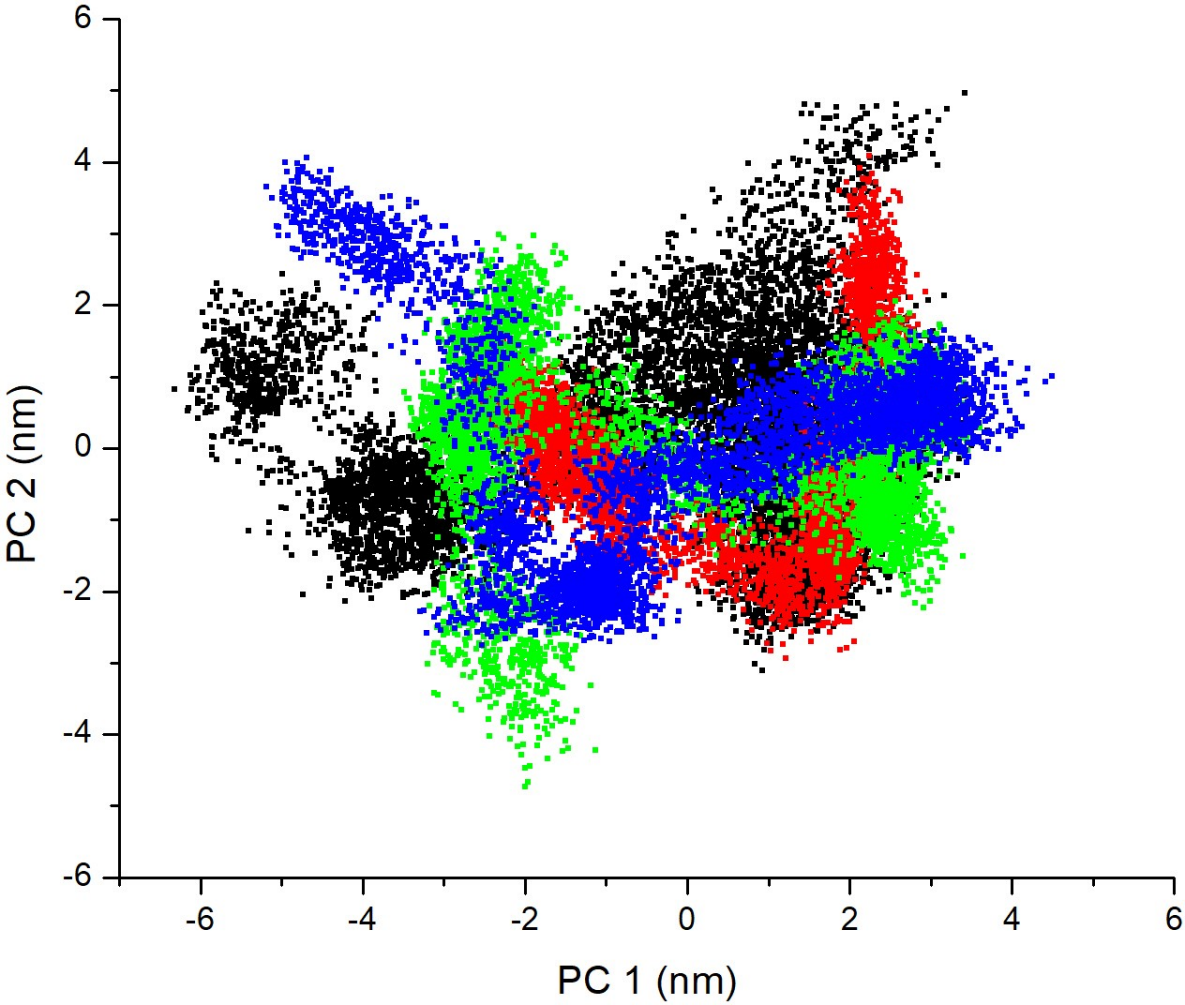
Principal component analysis. Projection of motion of Apo-LdMPK4 in black; ATP complex in red; GEN complex in green; CHY complex in blue. The rigidity of the ligand bound forms over the apo-enzyme can be analyzed by the smaller area covered by ligand complexes.

Principal component analysis (PCA) analysis was computed to extract large scale motions essential for the activity of the protein. PCA was thus done for apo-LdMPK4 and LdMPK4 complexes with ATP, GEN, and CHY. The first 10 residues were removed for analysis due to their non-availability of structural information as discussed in RMSF fluctuations. Covariance matrix was calculated after removing translational and rotational motion using gmx covar. The covariance matrixes of backbone C alpha atoms were then diagonalized to obtain the eigenvectors and eigenvalues [56, 57]. The first two projections (PC 1 and PC 2) with the highest eigenvalues were considered as they accounted for 80–90% of the collective motions of the C alpha backbone atoms. It was also analysed to be confined within the first four eigenvectors. The 2D plots of PC1 and PC2 of each of the LdMPK4-ligand complexes and apo-LdMPK4 are shown in Fig 5. The PCA analysis of ATP, GEN and CHY complex shows the rigidity as the area covered is much smaller and clustered closely as compared to the free enzyme.

#### Hydrogen bond interaction

**Fig 6:**
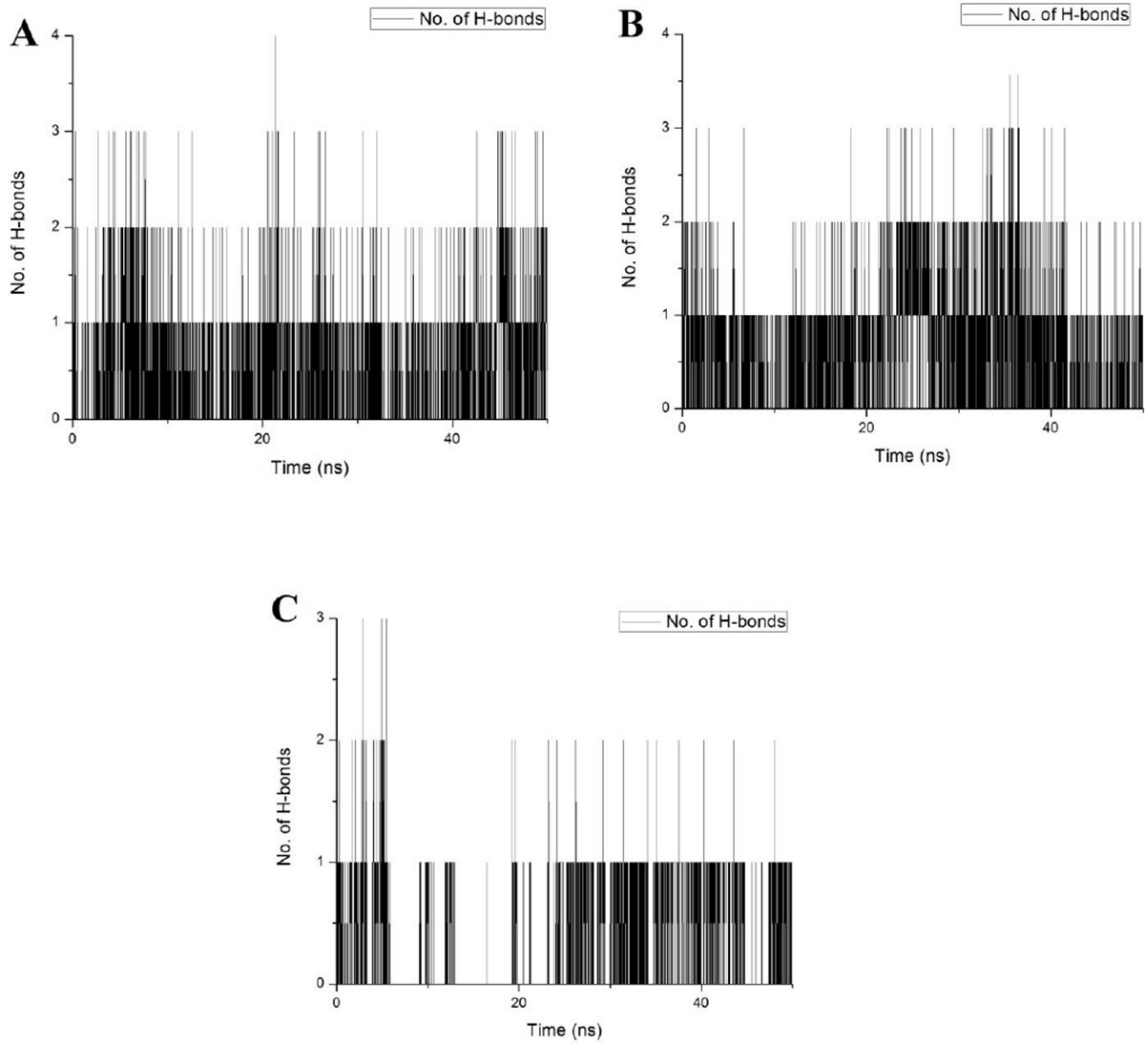
Hydrogen bond analysis. Hydrogen bond formation between the LdMPK4 and (A) ATP, (B) GEN and (C) CHY during the course of simulation is represented. Intermittent hydrogen bonds in CHY is evident in (C) but the ligand is not extruded from the binding cavity over 50ns production period.

The hydrogen bond analysis between LdMPK4 and the ligands were calculated using gmx hbond. Fig 6 shows the H-bond interaction between LdMPK4 and ligands over the period of 50 ns simulation period. The active site Lys59 was analysed for hydrogen bonding with all the ligands. ATP formed hydrogen bonding with Phe38, Gly42 and Lys59 continuously, thereby contributing majorly wherein Gly37, Gly39, Cys41, Arg73, and Asp174 formed intermittent hydrogen bonds (Fig 6A). ATP significantly forms continuous hydrogen bonds with active site Lys59 and the phosphate group was found to be aligned favourably towards the same while Ser160 was found vice. The amide group of Gly37, Gly39 and Cys41 forms intermittent hydrogen bonds with ATP along with amino and carboxyl group of Arg73 and Asp174 respectively. The adenosine and ribose part of the ATP is thus stabilised by these amino acid residues. In GEN, the H-bond formation is mainly concentrated between Phe38, Cys41, and Lys59 with Lys59 less preferred. Gly37, Gly39, Gly42, Arg73, and Asp174 also formed hydrogen bonds non-continuously over the period of 50 ns (Fig 6B). These interactions mainly involved the bond formation between the hydroxyl groups of GEN and amide, amino and carboxyl groups of amino acid residues. No hydrogen bonds were observed with Ser160 and Arg60 as observed in docking. The Ser160 was observed to be aligned unfavourably to the ligand molecule. In the case of CHY molecule, the hydrogen bond formation was analysed to be with Phe38, Cys41, Gly42, and Lys59. The hydrogen bonds were sporadic with Lys59 and no other amino acids residues in the active site formed hydrogen bonds (Fig 6C). Among the predicted active site residues, Glu77, Met81, Tyr114, Asp115, Thr116, Asp117, Arg120, Ser160, Asn161, Leu163, Cys173, and Phe175 did not form any H-bonds with any of the ligands. The hydrogen bonds even though lesser was found to be stable through electrostatic and van der Waals forces. GEN and CHY were stable within the binding site over the period of 50 ns though continuous and stable hydrogen bonds weren’t observed for CHY. Binding affinities of GEN and CHY were therefore analysed and compared with ATP by calculating the binding free energy between LdMPK4 and GEN/CHY contributed by electrostatic and van der Waals forces.

### Binding free energy calculation and residue-based decomposition

The free energy calculations were studied using g_mmpbsa. The affinity of the protein and the ligand in the docked complexes were analysed based on the MD trajectory of each protein-ligand system. The binding free energy (ΔG_bind_) and the individual components were calculated based on the following formula.

ΔGbind=EMM+Gsolv–TΔS=EvdW+Eelec+Gpolar+Gnon−polar−TΔS

The four individual energy components were compared for the three ligand complexes to analyse the contribution towards binding affinity. The ΔE_vdW_ component for was found to be -103.47 kJ/mol in ATP complex, -104.92 kJ/mol in GEN complex and -105.59 kJ/mol in CHY complex. The ΔE_vdW_ was found to be more or less similarly contributed in all the ligand complexes. In the case of ΔE_elec,_ the energy component was -3.58 kJ/mol in ATP complex, -10.13 kJ/mol in GEN complex and -11.38 in CHY complex. The electrostatic energy contribution was found to be significantly higher in GEN and CHY complex when compared to the ATP complex. When ΔG_polar_ was analysed, 60.08 kJ/mol in ATP complex, 46.50 kJ/mol in GEN complex and 49.81 kJ/mol in CHY was determined. The higher polar energy found in ATP complex with respect to the lower polar energies in GEN and CHY also contributed to the GEN and CHY inhibitors higher binding affinity. ΔGnon-_polar_ changes were insignificant in the complexes where -7.97 kJ/mol in ATP, -9.65 kJ/mol in GEN and -9.00 kJ/mol in CHY were found respectively.

The binding free energy ΔG_bind_ was found to be significantly different from the ATP substrate. The GEN and CHY complexes with LdMPK4 had better and similar binding energy when compared to ATP complex with LdMPK4. The difference in binding affinity was due to electrostatic, polar and non-polar energies in the inhibitor-protein complex. The presence of hydroxyl groups (OH) in these two molecules favoured better binding affinity when compared to natural substrate ATP. Both the molecules were also structurally similar with CHY being devoid of a hydroxyl group compared to GEN. This may be one of the reasons for reduced binding energies and stability of CHY over GEN. This significant ΔG_bind_ difference suggests that the GEN and CHY and their hydroxyl groups qualifies as inhibitor compounds competitive to ATP molecule.

**Fig 7:**
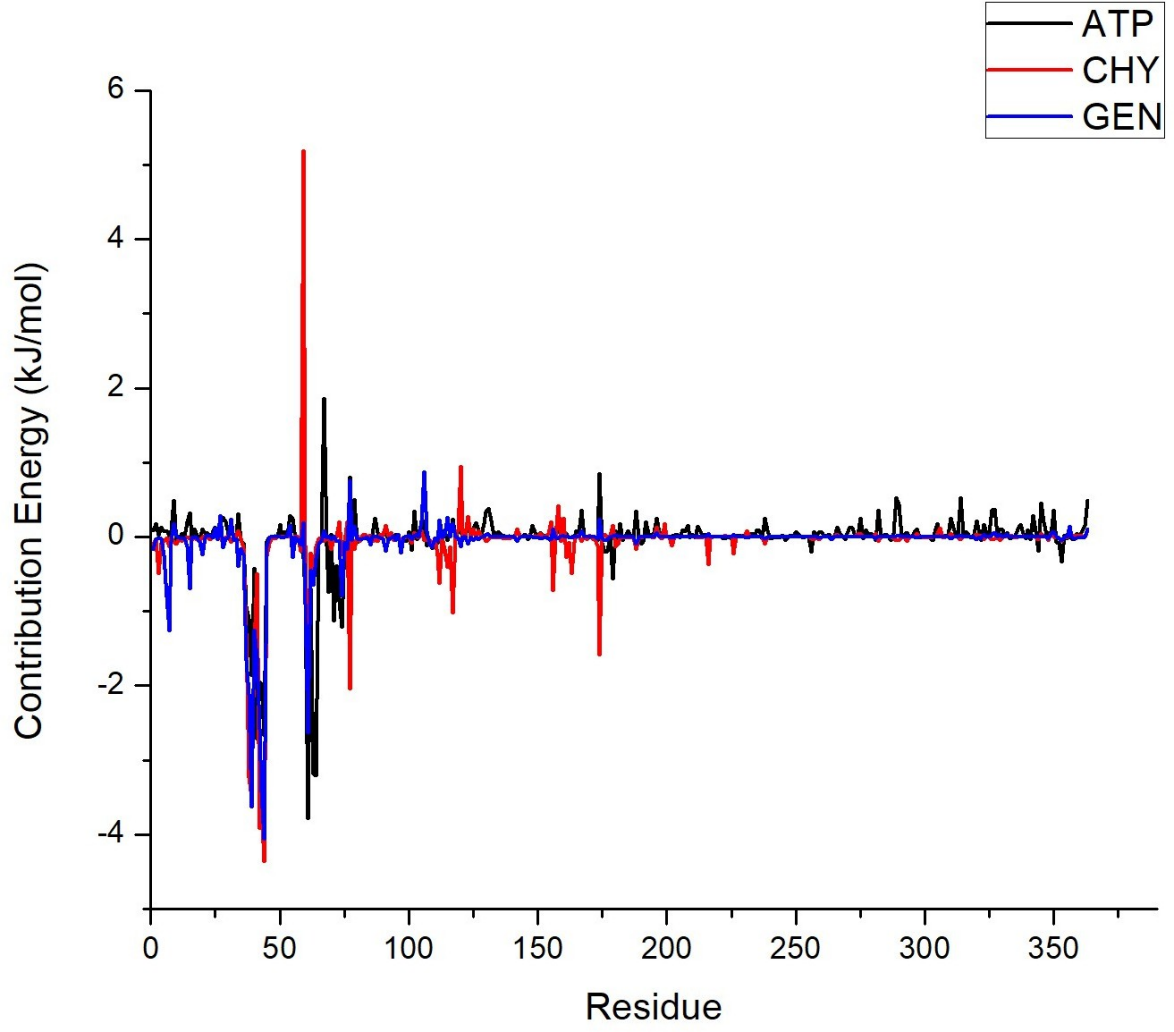
Binding energy calculation of ligands. Free binding energy (ΔG) contribution of 363 amino acid residues over the production period. ATP interaction with LdMPK4 enzyme residues in black; GEN in blue; CHY in red; interaction with enzyme LdMPK4. All the residues that contributed to various energy components ranged within most of the predicted active residues.

Energy based decomposition of the inhibitor-protein complexes was performed to understand the singular contribution of each and every residue in the active site [58, 59]. The stability of the complex based on the residues was investigated for interactions other that H-bonds. In the ATP complex, the major contributions were from residues Phe38, Gly39, Cys41, Gly42, Thr43, Val44, Leu61, Arg63, Val64, and Ile74. Comparing GEN complex, the favourable contributions were from Leu7, Gly37, Phe38, Gly39, Ala40, Cys41, Gly42, Thr43, Val44, Arg60, Leu61, and Ile74. The negative contributions in CHY came from the residues Phe38, Gly39, Ala40, Gly42, Thr43, Val44, Leu61, Glu77, and Asp117. In the GEN and CHY complexes, these residues with most of them belonging to the predicted active site residues maintain the stability of the complexes in the binding cavity. Even though there were intermittent hydrogen bond formations, the inhibitors were maintained in the binding cavity by electrostatic, polar and nonpolar interaction of the residues as seen in Table 4. There were many other residues in the binding cavity that formed minor negative energy contributions as represented in Fig 7. The total binding free energy formed by GEN and CHY complex is greater than ATP free energy formed; moreover, GEN had an upper hand over CHY in the binding free energy too. This strongly suggests that there exist strong interactions in terms of other energy components than hydrogen bonding therefore strongly substantiating the point that GEN and CHY can be strong inhibitors of LdMPK4 enzyme.

**Table 4:** Binding free energy (kJ/mol) calculation for MAPK ligand complexes with GEN, CHY and ATP as determined by g-mmpbsa.

Component	Ligand		
	ATP	GEN	CHY
**E**_vdW_ (kJ/mol)	-103.47	-104.923	-105.592
**E**_elec_(kJ/mol)	-3.588	-10.134	-11.382
**G**_polar_(kJ/mol)	60.086	46.500	49.818
**G**_non-polar_(kJ/mol)	-7.973	-9.653	-9.007
Δ**G**_bind_(kJ/mol)	-54.946	-78.211	-76.164

## Conclusion

The in-silico study concludes that the two molecules GEN, an isoflavone and CHY, a flavone has been identified likely to be potential inhibitors of LdMPK4 enzyme. The pharmacological properties of these inhibitors also suggest their qualification as a lead molecule for drug studies. Docking studies had hypothesized that these molecules GEN and CHY, could act as inhibitors of LdMPK4 enzyme. The molecular dynamic simulation and free energy calculations were analysed along with the natural substrate ATP. The RMSD from the three compounds and from the apo-enzyme displayed stable backbone throughout the simulation period. RMSF analysis also revealed minor fluctuations in the active site residues in all three complexes. R_g_ and SASA analysis predicted a compact complex protein structure and interactions between the complex structure and solvents also found small conformational changes of the ligands in the binding cavity, the results project the comfortability of the ligands in the binding cavity. Though hydrogen bonding revealed continuous bonding in GEN and ATP complex with active site Lys59 and other binding cavity residues, CHY complex displayed intermittent binding. The intermittence in the inhibitor-protein complex was counterbalanced by the electrostatic, polar and non-polar energy interactions between them. The stable energy interactions over the period of 50 ns indicated that the overall protein structure remained stable and the presence of ligand molecules in the binding cavity did not hinder the same. The presence of OH groups in the ringed molecules contributed to the higher binding affinity of the molecules compared to ATP as presented by the MMPBSA calculations. The results from the following study evidently indicate that the lead compounds GEN and CHY can emerge as potential inhibitors of LdMPK4 enzyme.

## Supporting information

S1 Fig Validation of modelled LdMPK4 structure using (A) ProSA analysis server with overall model quality z-score of -6.46 (B) Ramachandran Plot showing 7.1% residues in the outlier region.
(TIF)

S2 Fig Multiple sequence alignment. Multiple sequence alignment of modelled LdMPK4 modelled (s001) against Human (5MTX), *P*. *bergheri* (3N9X), *L*. *donovani* (4QNY) and *T*. *gondii* (3RP9). Color coding of residues: alpha helix(red), beta-strand(blue). Consensus secondary structure elements of modelled MAPK4 is represented below along with consensus amino acids in bold and upper case (aliphatic(l), hydrophobic (h), aromatic (@), alcohol (o), polar (p), tiny (t), small (s), bulky (b), positive (+), negative (-) and charged (c).
(TIF)

S3 Fig Structural analysis of inhibitors. Chemical Structure and information pertaining to proposed inhibitors (A) Genistein and (B) Chrysin.
(TIF)

S4 Fig Docked poses of ligands. Superimposed ligands ATP (Maroon), GEN (Magenta) and CHY (Blue). It is visible that all the docked ligands are found to share the same binding cavity.
(TIF)

S5 Fig Secondary structure of modelled protein. Secondary structure prediction of LdMPK4 enzyme observed using DSSP during the MD simulation.
(TIF)
