# Supplementary figures and images for: Identification of lead molecules against potential drug target protein MAPK4 from L. donovani: An in-silico approach using docking, molecular dynamics and binding free energy calculation
Source: PLoS One. 2019 Aug 19;14(8):e0221331. doi: 10.1371/journal.pone.0221331 (PMC6699710; doi:10.1371/journal.pone.0221331)

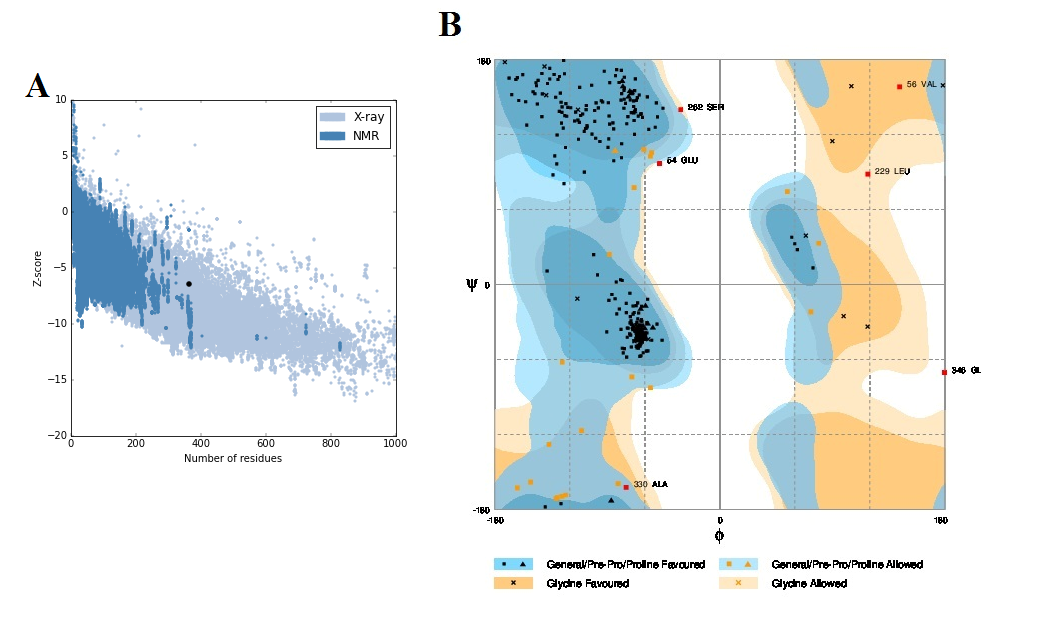

Supplement: S1 Fig — Validation of modelled LdMPK4 structure using (A) ProSA analysis server with overall model quality z-score of -6.46 (B) Ramachandran Plot showing 7.1% residues in the outlier region. (TIF) [file pone.0221331.s001.tif]

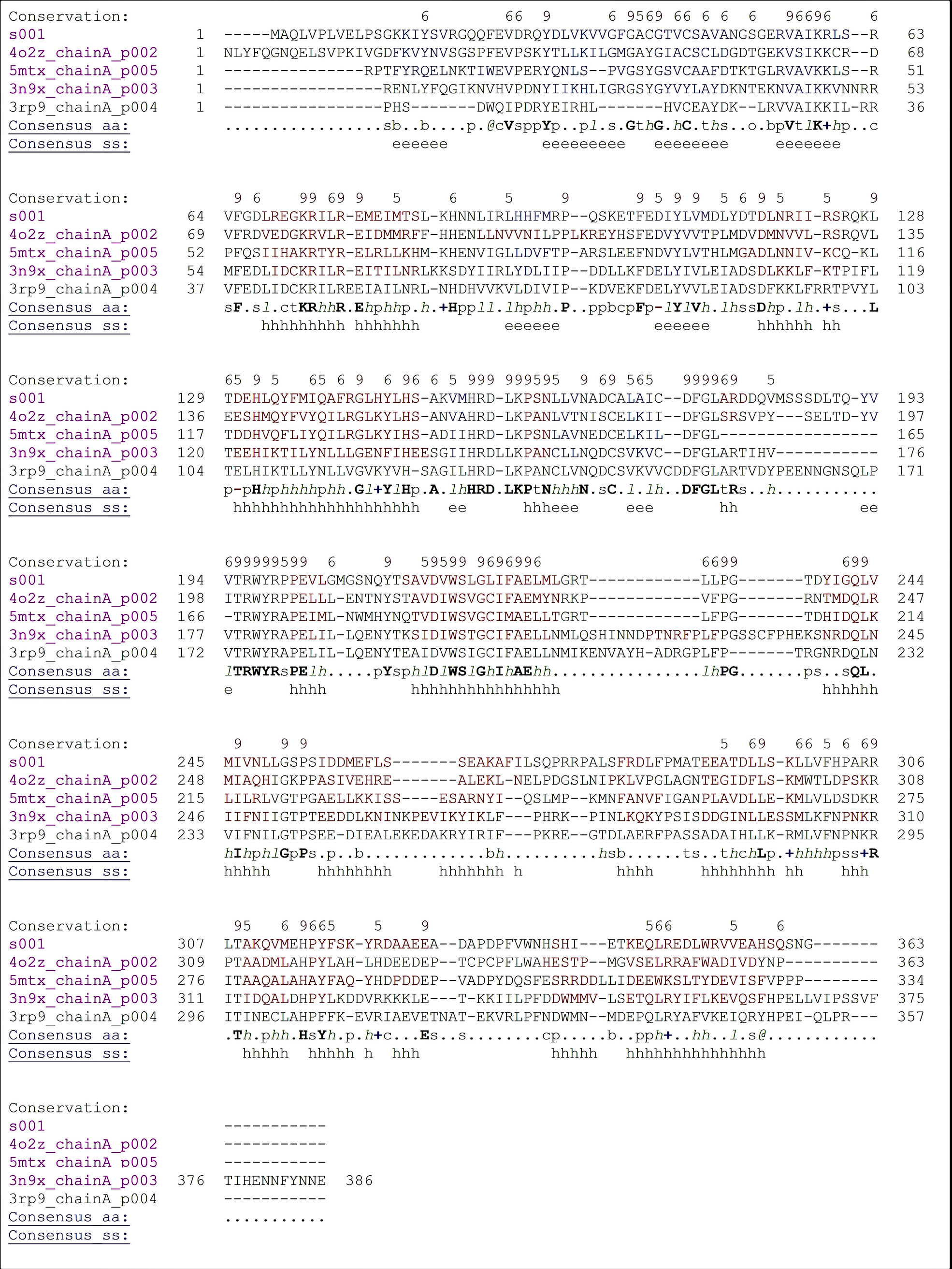

Supplement: S2 Fig — Multiple sequence alignment of modelled LdMPK4 modelled (s001) against Human (5MTX), P. bergheri (3N9X), L. donovani (4QNY) and T. gondii (3RP9). Color coding of residues: alpha helix(red), beta-strand(blue). Consensus secondary structure elements of modelled MAPK4 is represented below along with consensus amino acids in bold and upper case (aliphatic(l), hydrophobic (h), aromatic (@), alcohol (o), polar (p), tiny (t), small (s), bulky (b), positive (+), negative (-) and charged (c). (TIF) [file pone.0221331.s002.tif]

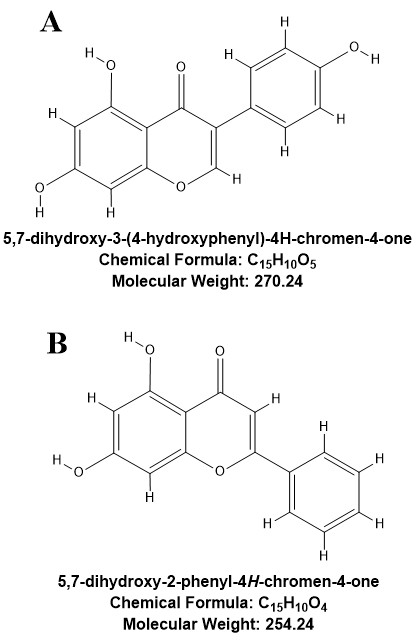

Supplement: S3 Fig — Chemical Structure and information pertaining to proposed inhibitors (A) Genistein and (B) Chrysin. (TIF) [file pone.0221331.s003.tif]

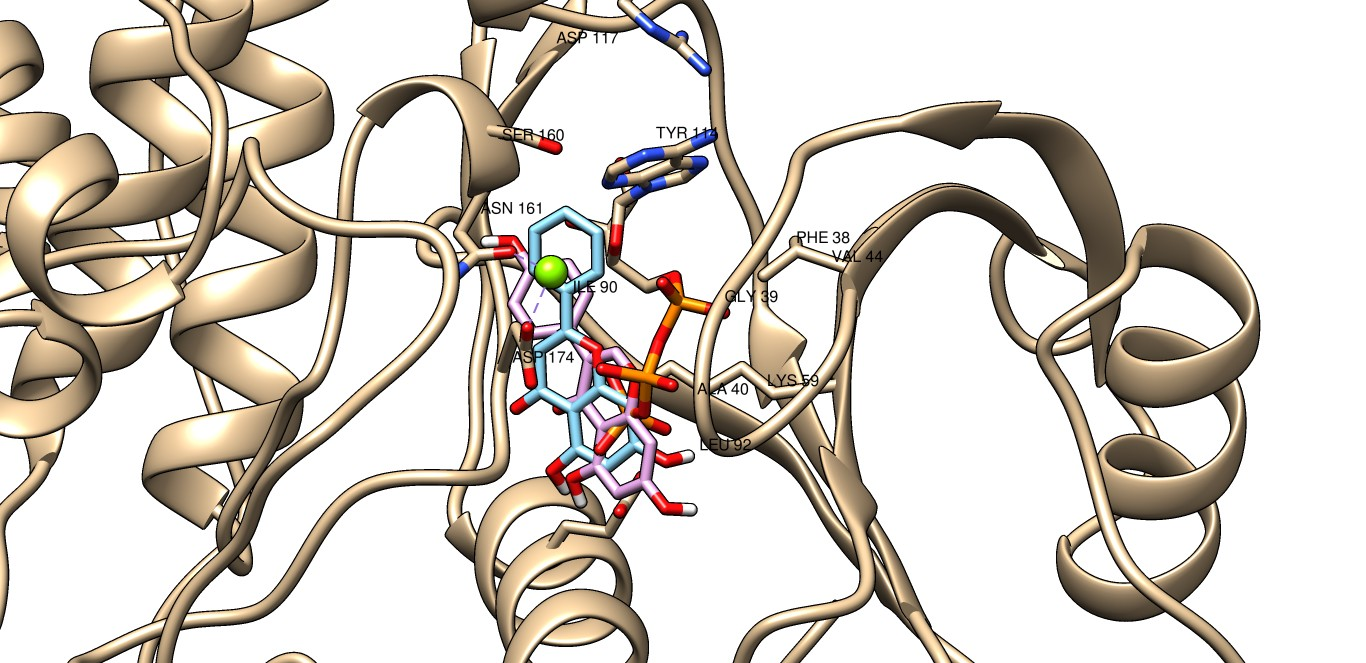

Supplement: S4 Fig — Superimposed ligands ATP (Maroon), GEN (Magenta) and CHY (Blue). It is visible that all the docked ligands are found to share the same binding cavity. (TIF) [file pone.0221331.s004.tif]

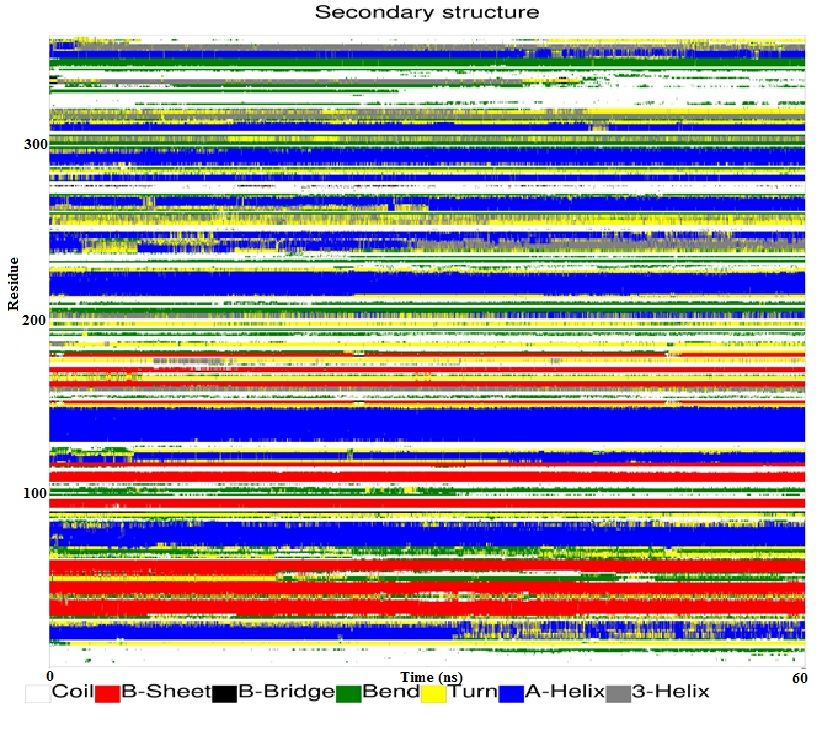

Supplement: S5 Fig — Secondary structure prediction of LdMPK4 enzyme observed using DSSP during the MD simulation. (TIF) [file pone.0221331.s005.tif]
